# Recent advances in managing septal defects: atrial septal defects

**DOI:** 10.12688/f1000research.11844.1

**Published:** 2017-11-22

**Authors:** P Syamasundar Rao, Andrea D Harris

**Affiliations:** 1 University of Texas-Houston McGovern Medical School, Children Memorial Hermann Hospital, Houston, USA

**Keywords:** Atrial Septal Defects, shunt, OSTIUM SECUNDUM

## Abstract

The purpose of this review is to discuss the management of atrial septal defects (ASD), paying particular attention to the most recent developments. There are four types of ASDs: ostium secundum, ostium primum, sinus venosus, and coronary sinus defects. The fifth type, patent foramen ovale—which is present in 25 to 30% of normal individuals and considered a normal variant, although it may be the seat of paradoxical embolism, particularly in adults—is not addressed in this review. The indication for closure of the ASDs, by and large, is the presence of right ventricular volume overload. In asymptomatic patients, the closure is usually performed at four to five years of age. While there was some earlier controversy regarding ASD closure in adult patients, currently it is recommended that the ASD be closed at the time of presentation. Each of the four defects is briefly described followed by presentation of management, whether by surgical or percutaneous approach, as the case may be. Of the four types of ASDs, only the ostium secundum defect is amenable to percutaneous occlusion. For ostium secundum defects, transcatheter closure has been shown to be as effective as surgical closure but with the added benefits of decreased hospital stay, avoidance of a sternotomy, lower cost, and more rapid recovery. There are several FDA-approved devices in use today for percutaneous closure, including the Amplatzer® Septal Occluder (ASO), Amplatzer® Cribriform device, and Gore HELEX® device. The ASO is most commonly used for ostium secundum ASDs, the Gore HELEX® is useful for small to medium-sized defects, and the cribriform device is utilized for fenestrated ASDs. The remaining types of ASDs usually require surgical correction. All of the available treatment modes are safe and effective and prevent the development of further cardiac complications.

## Introduction

Septal defects are among the most common types of congenital heart defects (CHDs) and typically present with left to right shunts. The sequelae of such defects are related to the size of the defect, amount of shunting, duration of shunting, and reactivity of the pulmonary vascular bed. Nearly 50% of these defects resolve spontaneously or may be treated with medical or expectant management, while the other half will require intervention in the cardiac catheterization laboratory or in the operating room. While surgical repair has been the mainstay of addressing these defects, more recently transcatheter (percutaneous) and hybrid approaches have been used to treat these defects effectively. In these reviews, we will present a classification of the atrial septal defects (ASDs), ventricular septal defects (VSDs), and atrioventricular septal defects (AVSDs), indications for closure, and a discussion of surgical and transcatheter closure approaches as well as the benefits and risks of transcatheter versus surgical intervention. In this paper, only ASDs will be addressed, while VSDs and AVSDs will be dealt with in a subsequent paper.

ASDs produce left to right shunt, since the left atrial pressure is greater than that in the right atrium. Such shunting causes volume overloading of the right heart. Whereas such shunts are usually well tolerated during infancy and childhood, exercise intolerance and arrhythmias may manifest in late childhood and adolescence and there is a greater risk of developing pulmonary vascular obstructive disease in adulthood. Consequently, these defects are clinically important.

Four major types of ASDs are recognized; these are ostium secundum, ostium primum, sinus venosus, and coronary sinus defects ^1– 3^. The fifth type, patent foramen ovale (PFO), is present in nearly one-third of the normal population and may be considered a normal variant. While left to right shunts across the PFO is not problematic, they are important in the presence of other CHDs or if they are the source of right to left shunt producing paradoxical embolism with resultant stroke/transient ischemic attacks or other problems, including migraine, Caisson’s disease, and platypnea-orthodeoxia syndrome. Because of limitations of space, further discussion of PFO and its closure will not be undertaken in this review.

## Indications for ASD closure

Closure of moderate to large ASDs is recommended, even in the absence of symptoms at presentation. The reasons for such recommendations are to 1) prevent the development of pulmonary vascular obstructive disease in adulthood, 2) decrease the chances of supra-ventricular arrhythmias later in life, and 3) preclude the development of symptoms during adolescence and adulthood. Elective occlusion at four to five years of age is usually recommended. Closure during infancy is not required unless the infant is symptomatic.

Small defects (<5 mm) are likely to spontaneously close and do not need occlusion. Evidence for right ventricular volume overloading (dilatation of right atrium and right ventricle with flat or paradoxical interventricular septal motion) by echocardiogram is used by most cardiologists as an indication for closure. If cardiac catheterization is performed, pulmonary to systemic flow ratio (Qp:Qs) >1.5 is an indication for closure.

The above are developed largely to address secundum ASDs. Similar criteria are used for closure of ostium primum, sinus venosus, and coronary sinus ASDs. The degree of mitral insufficiency is an additional consideration in ostium primum ASDs.

While some early studies ^4– 6^ argued that there is no major benefit of ASD closure in adults, more recent studies showing a high incidence of major cardiovascular events in older adult subjects with unrepaired ASD ^7^, safety and effectiveness with high event-free survival rates in adults subsequent to surgical closure of ASD ^8, 9^, improved cardiac function following ASD closure in adults ^10^, and improved functional capacity following ASD closure ^11^ led to the conclusion that all adult patients with evidence of right ventricular volume overload should have their ASD closed at presentation ^12, 13^.

## Ostium secundum ASDs

ASDs constitute 10% of all CHDs. There is deficiency of the atrial septal tissue in the region of the fossa ovalis. These defects may be small, medium, or large in size and oval, circular, or irregular in shape. The majority are single defects; however, multiple defects and fenestrated defects may occasionally be seen. Left to right shunt across the ASD produces dilatation of the right atrium, right ventricle, and main and branch pulmonary arteries. Pulmonary vascular obstructive disease does not manifest until adulthood, and even then it is rare, at least until late adulthood.

### Management

The treatment of ASD patients is mostly dependent on the age at presentation, existence of symptoms of congestive heart failure, and the degree of the shunt. In an occasional infant with congestive heart failure, anti-congestive measures (diuretics and digoxin) should be provided. Subsequently, surgical or trans-catheter closure of the defects should be performed. While it is feasible to percutaneously close the ASD, even in infants, because spontaneous closure of even large ASDs can occur, the ASD should not be closed in the small asymptomatic child. Such children may be followed by periodic echocardiography, giving an opportunity for spontaneous closure. SBE prophylaxis is not necessary and activity restriction is not recommended for these patients.

***Surgery.*** Subsequent to the description of cardiopulmonary bypass techniques for the surgical closure of ASDs by Gibbon, Lillehei, and Kirklin in the 1950s, surgery has become a customary treatment for ASDs. Under general anesthesia, a median sternotomy or a right submammary incision is performed and the aorta and vena cavae are cannulated to place the patient on cardiopulmonary bypass. Right atriatomy exposes the defect. Closure of the defect is done by either approximating the defect margins with suture material or using a pericardial or Dacron patch, based on the size of the defect and the surgeon’s preference. Primary closure is generally reserved for small defects, while patch closure is used for larger defects. Primary closure allows for the possibility of suture rupture and reopening of the defect, while patch closures may be associated with residual shunt. More recently, minimally invasive and robotic-assisted surgery has been used with good results ^14, 15^.

Surgical closure of ostium secundum ASDs is considered safe and effective with negligible (<1%) mortality. However, the morbidity related to sternotomy/thoracotomy and cardiopulmonary bypass and the potential for postoperative complications are unavoidable. Additional disadvantages of surgery are the associated expense and residual scar and psychological trauma to the patient and/or their parents. Presumably because of these reasons, trans-catheter methods have been developed, as reviewed elsewhere ^16– 20^. Currently, surgery is mainly reserved for ASDs with poor septal rims deemed difficult to close with trans-catheter techniques or unsuccessful percutaneous attempt to close the defect. In addition, if the repair of other associated defects is anticipated, surgical closure of the ASD during that surgery may be accomplished concurrently.

***Percutaneous closure.*** King and Mills initially described the transcatheter occlusion of ASDs ^21^. Shortly thereafter, Rashkind and Cuaso presented another type of device to close ASDs ^22^. A large number of other devices have subsequently been developed over the last four decades, as reviewed elsewhere ^16– 20, 23, 24^, and include: modified Rashkind’s device with six stainless steel arms with every other arm impregnated with a miniature “fish” hook, modified Rashkind device with double-disc, clamshell occluder, original buttoned device, atrial septal defect occluding system (ASDOS), second- and third-generation buttoned devices, Monodisk device, Das-Angel-Wing device, modified Rashkind PDA umbrella device, centering buttoned device, inverted buttoned device, Amplatzer ^®^ Septal Occluder (ASO), CardioSEAL ^®^ device, STARFlex device, fourth-generation buttoned device, Sideris’ wireless device, Sideris’ transcatheter patch, Gore HELEX ^®^ Septal Occluder, centering-on-demand buttoned device, fenestrated Amplatzer ^®^ device, hybrid buttoned device, cribriform device to occlude multiple defect, BioSTAR ^®^, nanoplatinum-coated Amplatzer ^®^ device, Solysafe septal occluder device, Bio-STAR device, BioTREK device, Occlutech septal occluder, ATRIASEPT I-ASD device, ATRIASEPT II-ASD and ULTRASEPT, the pfm ASD-R device, biodegradable polycaprolactone occlusion device, Nit-Occlud ASD-R ^®^ (NOASD-R) device, Lifetech Cera, and perhaps others. The majority of the devices had initial device closure attempts in animal models followed by clinical trials in human subjects with local IRB approval, CR Mark approval in Europe, or under FDA-approved clinical trials in the USA. Feasibility, safety, and effectiveness of occluding the ASD have been demonstrated for most of the devices. However, as of this time, the ASO, Amplatzer ^®^ Cribriform device, and Gore HELEX ^®^ are the only devices that have received approval for clinical use by the FDA in the USA. The Occulutech Septal Occluder, which has design similarities to the ASO, is widely used outside of the USA.

**Amplatzer ^®^ Septal Occluder**

The ASO consist of two discs created from 0.004” to 0.007” Nitinol (nickel–titanium compound) wire with shape memory and with Dacron polyester patches sewn into each disc; the discs are connected by a 4 mm long waist which sits within the defect. The disc on the left atrial side is slightly larger than the one on the right. Transesophageal echocardiography (TEE) or intracardiac echocardiography (ICE) is used to monitor device deployment. Most cardiologists use static balloon sizing of the ASD using NuMed PTS or AGA Amplatzer sizing balloons. However, the senior author does not recommend or perform balloon sizing routinely but relies on the TEE (or ICE) sizing utilizing the thick margins of the ASD, excluding the flail margins, a method suggested by Carcagnì and Presbitero ^25^. An ASO that is 1 to 2 mm larger than the diameter of the ASD is chosen for implantation. The device is deployed into the left atrium, and, after ensuring that the device does not impinge on any structures, the larger left atrial disc is pulled against the atrial septum, the waist of the device is delivered into the defect, and the right atrial disc is then deployed on the right side of the septum. Once the device is positioned, careful echocardiographic views are required to ensure the discs of the device are not impinging on any structures and in particular the left atrial disc is not impinging on the anterior leaflet of the mitral valve. The device is self-centering within the defect and is retractable into the delivery sheath to allow for repositioning as needed. A “mushroom” appearance of the device indicates that the device is too large for the defect and, in such cases, the device is removed and replaced with a smaller device. Too large a device within the defect increases the risk of erosion of the atrial wall or the aorta, as will be reviewed in the Complications section. Once the device position is considered satisfactory, the device cable is moved back and forth (so-called Minnesota Wiggle) and repeat TEE or ICE is performed. If this maneuver results in prolapse of the device into the atria, the device needs to be removed and replaced with a larger device and TEE/ICE repeated prior to release of the device and termination of the procedure.

Complex defects, namely large defects, small septal rims, multiple defects, and septal aneurysms are problematic, and suitable modifications in the technique should be embarked upon to guarantee success of the device implantation, as detailed elsewhere ^26, 27^.

Immediate and follow-up results of ASO implantations appear encouraging, with immediate complete closure rates varying from 62 to 96%, which improved to 83 to 99% at 6- to 12-month follow-up ^28^. Other studies in children ^29– 33^ and adults ^34– 36^ show similar results. We undertook the closure of 150 ostium secundum defects with ASO; small residual shunts were seen in two patients at the end of the procedure, but the shunts disappeared at 1- and 6-month follow-up visits, respectively. One patient developed complete heart block, requiring transvenous pacemaker insertion. No residual shunts were observed during a mean follow-up of 24 months ^37, 38^. In the USA, the ASO is becoming the device of choice based on the ease with which the device can be deployed, retrieved, and repositioned and the comfort that the device is approved by the FDA.

**Amplatzer ^®^ Cribriform Device**

The cribriform device is fabricated in a way that is similar to that of the ASO; it consists of two equal-sized discs connected with a thin waist ^39– 41^. The cribriform device is used for the closure of fenestrated ASDs. The device implantation is similar to that of the ASO, the delivery sheath should be positioned in the middle fenestration of the ASD, and the discs should cover the most peripheral fenestration to yield effective closure. The limited published information suggests satisfactory outcomes ^39– 41^.

**Gore HELEX ^®^ Septal Occluder Device**

The Gore HELEX ^®^ device consists of a single Nitinol wire covered by ultrathin expanded polytetrafluoroethylene (ePTFE) which forms two inter-connected discs when deployed. Unlike the ASO device, it is not self-centering and is only useful in small to moderate defects (up to 18 mm in diameter). The device is manufactured in 15 to 35 mm diameter sizes in 5 mm increments.

The procedure of implantation is similar to that alluded to in the ASO section and is described in detail elsewhere ^42^. In brief, the delivery catheter (green) is placed in the left atrium over a guide wire. The push-pinch-pull method is used to develop the left atrial disc and the disc is then withdrawn gently to engage the left side of the atrial septum while fluoroscopic monitoring and TEE or ICE is performed. Then the green catheter is withdrawn over the gray catheter until the mandrel (tan) engages the hub. The green catheter is then held steady while the gray catheter is advanced to place the right atrial disc on the right atrial side of the atrial septum, again using the push-pinch-pull method. Once the device’s position is confirmed by TEE or ICE, it is locked and then released.

A multicenter trial ^43^ demonstrated successful implantation in 87% of patients, with a 2.6% incidence of residual leaks at follow-up 1 year after device implantation. Other reports indicate similar results. Wire frame fractures were observed in 8% of patients. A more recent analysis of 435 patients in the USA revealed composite clinical success in 93% of patients at 12-month follow-up ^44^. The Gore HELEX ^®^ device is generally thought to be a suitable device for the closure of small to medium-sized defects.

### Complications associated with device closure

Device-associated complications include device dislodgement and embolization, residual shunts, wire fractures, and heart block, although these are rare. Migration of the device along with erosion of the aorta during follow-up was detected in 18 out of 15,900 (0.12% or 1 in 1,000) ASO implantations in a US study ^45, 46^. A similar prevalence (37 out of 35,000, 0.11%, or 1 in 1,000) in ASO implants worldwide was noted ^44– 47^. The data were examined by the Review Board and AGA Medical (AGA 2006); these reviews indicated that the device erosion may be associated with over-sizing of the device and recommended that a device size more than 1.5 times the TEE/ICE diameter size of the ASD should not be used for closure of the defect. A more recent analysis revealed that patients with aortic erosion are more likely to have oversized devices, deficient aortic or superior vena caval rims, large balloon size, large ASO, larger device size-static ASD diameter difference, smaller patient age:device size ratio, and smaller weight:device size ratio than are control subjects ^48^.

### Benefits and risks of transcatheter versus surgical intervention

A number of studies ^49– 56^ compared surgical with device closure; these studies suggest equal effectiveness, and the transcatheter closure of ostium secundum ASDs has been proven to be a safe and effective alternative to surgical closure. The benefits of avoiding thoracotomy/sternotomy, cardio-pulmonary bypass, and cardiotomy, a decreased period of hospitalization (one day versus three to four days), and fewer complications (complication rates of transcatheter intervention have been reported at 7.2%, while the surgical group is reported at 24%) are measurable. In addition, percutaneous closure is less expensive than surgery. Percutaneous closure of secundum ASDs utilizing various devices is currently a conventional practice in the majority of institutions offering state-of-the-art care to subjects with cardiac disease ^13, 57^.

## Ostium primum ASDs

Ostium primum ASDs belong to the collection of defects called AVSDs; they are usually large in size and are located in the anterior portion of the lower part of the atrial septum. A cleft in the anterior leaflet of the mitral valve is almost always present and produces mitral insufficiency. A cleft in the septal leaflet of the tricuspid valve may be seen in some patients. These defects, formerly known as partial endocardial cushion defects, are also called partial AVSDs. Ostium secundum ASD, PFO, or persistent left superior vena cava draining into the coronary sinus may also coexist. The outflow tract of the left ventricle is long and narrow and is described as a goose-neck deformity, and sometimes left ventricular outflow tract obstruction may be present. Dilatation of the right heart structures, similar to that described for ostium secundum ASDs, is present. Left atrial and left ventricular dilatation may be seen if mitral insufficiency is moderate to severe in degree.

### Management

The indications for repair are similar to those listed for the closure of ostium secundum ASDs, and the usual recommended age for surgical repair is three to five years. However, in the presence of congestive heart failure secondary to mitral insufficiency, surgical repair may be performed at presentation following control of the heart failure by medical management.

***Medical management.*** Though rare, congestive heart failure is seen with moderate to severe mitral insufficiency and should be treated with anti-congestive measures (afterload reducing agents, diuretics, and digoxin). Once the symptoms of congestive heart failure are controlled, surgical repair should be undertaken. SBE prophylaxis is recommended in the presence of significant mitral valve abnormalities.

***Surgical management.*** As mentioned in the preceding section, the conventional treatment option for ostium secundum ASDs is percutaneous occlusion; however, surgical correction is required for ostium primum ASDs. Percutaneous occlusion is not feasible in patients with ostium primum ASDs because there is no inferior septal rim and there is the need to address mitral valve cleft and mitral insufficiency. A median sternotomy incision is made under general anesthesia, and the patient is placed on cardiopulmonary bypass by cannulating the aorta and vena cavae. Right atriatomy will allow the exposure of the ASD and mitral valve. Interrupted suture material is used to close the mitral valve cleft. Additional reparative procedures (such as annuloplasty) to deal with the detected mitral valve abnormalities are undertaken. The atrial defect is then closed using an autologous pericardial patch. Rarely, other prosthetic materials such as Dacron or Gore-Tex are used. If ostium secundum ASDs or a PFO is present, they should also be closed at the same time.

The results of surgery are usually good, with a mortality rate of less than 3%. Poor results are likely in patients with severe mitral insufficiency, failure to thrive, and congestive heart failure. Late reoperations for mitral regurgitation, mitral stenosis, or subaortic stenosis may be required in 10 to 15% of patients.

## Sinus venosus ASDs

Sinus venosus ASDs constitute 5 to 10% of all ASDs and are of two types. The majority of defects are situated in the posterior superior portion of the inter-atrial septum, often overriding the orifice of the superior vena cava. Infrequently, the ASD may be positioned in the inferior-posterior part of the atrial septum, overriding the inferior vena cava. These ASDs are often seen in association with anomalous pulmonary venous connection. The right upper pulmonary veins (rarely the veins from the entire right lung) are connected to the superior vena cava or to the right atrium near the cavo-atrial junction. The dilatation of right heart structures is comparable to that depicted for the ostium secundum and primum ASDs.

### Management

The indications for intervention are, again, similar to those described in the ostium secundum ASDs. While device closure is a standard therapy in the management of ostium secundum ASDs, such an approach may not feasible in sinus venosus ASDs because of the lack of superior rim and the requirement for redirecting the anomalous pulmonary vein(s). However, some attempts to circumvent such problems by the implantation of a covered stent to direct the superior vena caval flow into the right atrium while occluding the atrial defect and redirecting the anomalous pulmonary vein into the left atrium have been made (Lin CH and Breinholt JB, personal communication, 2017). Further experimentation/research with such percutaneous approaches is warranted.

At the present time, surgical repair is the treatment of choice. Closure of the ASD along with diversion of the anomalous right pulmonary vein(s) into the left atrium are performed under cardiopulmonary bypass. Sometimes this may entail constructing a tunnel with an autologous pericardial patch along with enlargement of the superior or inferior vena cava, as the case may be. The results are generally good, with rare superior vena caval or pulmonary venous obstruction.

## Coronary sinus ASDs

Coronary sinus ASDs are the most rare of the ASD types. These are defects in the inferior and anterior part of the atrial septum at the anticipated site of the opening of the coronary sinus. These defects are frequently related to a persistent left superior vena cava and un-roofing of the coronary sinus, a complex originally described as Raghib syndrome ^58^. These defects may also be seen in patients with asplenia syndrome. Dilatation of right heart structures is similar to that portrayed for the other ASDs.

### Management

Surgical repair with patch closure of the defect, leaving the coronary sinus opening in the left atrium, is the usual approach ^59^. These defects are typically not amenable to percutaneous closure. Nonetheless, small defects may be amenable to percutaneous closure ^60^.
